# Correction: *“Africans*, *we know how to adapt indeed”*: Adaptations to family planning and reproductive health services in humanitarian settings in Nigeria during the COVID-19 pandemic

**DOI:** 10.1371/journal.pgph.0004037

**Published:** 2024-12-02

**Authors:** Emily Evens, Ashley Ambrose, Bamidele Bello, Kate Murray, Nadia Tefouet, Adesegun Fatusi, Bridget Nwagbara, Mercy Riungu, Tijani Maji, Hadiza Khamofu, Jean Christophe Fotso, Ndola Prata

The Data Availability statement for this paper is incorrect. The correct statement is: The datasets used for this study are available on the Harvard Dataverse and can be accessed using this link: https://doi.org/10.7910/DVN/H6MUWB.
