# Supplementary material for: “Africans, we know how to adapt indeed”: Adaptations to family planning and reproductive health services in humanitarian settings in Nigeria during the COVID-19 pandemic
Source: PLOS Glob Public Health. 2023 Jul 11;3(7):e0002104. doi: 10.1371/journal.pgph.0002104 (PMC10335652; doi:10.1371/journal.pgph.0002104)
Supplement: Attachment — Submitted filename: PLOS Reviewer Comments and Responses.docx [file pgph.0002104.s001.docx]

Please use the space provided to explain your answers to the questions above. You may also include additional comments for the author, including concerns about dual publication, research ethics, or publication ethics. (Please upload your review as an attachment if it exceeds 20,000 characters)

Reviewer #1: It is good to see data on what happened to SRH service provision in humanitarian settings during Covid. I have a few minor comments – mostly questions of clarity.

- **You mention lockdowns in some states. Can you provide any detail for what the Covid restrictions were in the 2 states where the study took place (Borno and Cross River)? For example, how long were lockdowns?** COVID restrictions included total stay at home for all, no interstate movements, no gatherings of more than 5 people, and only emergencies allowed in health facilities. A national lockdown was ordered on March 30, 2020, with additional states declaring lockdowns during April 2020. In Borno and Cross River states, restrictions included closure of borders to avoid movement in or out of the state, closure of schools and churches, limiting the number of passengers on public transportation, limiting gatherings to only 5 people in a space at a time, closure of government offices with exceptions or essential services like and law enforcement. There is variation in the length of COVID restrictions with some lockdowns lasting 2 to 3 weeks and other restrictions being continued for longer. Additional information has been included in the Background section.
- **Are all of the health facilities included in this study public health facilities? Or are some NGO-run?** The health facilities in the IHANN program in Borno state are NGO-run, while the health facilities in the UNHCR program in Cross River state are public health facilities. This has been added to Table 1.
- **Table 1: I’m not sure why you have FP as well as Referrals for FP. It might be helpful to indicate which methods are available through direct service delivery vs via referral?** We agree that this could be confusing. Methods offered through direct service delivery include condoms, oral contraceptive pills, injectables and implants. Referrals are made for IUCD and surgical methods such as Bilateral Tubal Ligation and vasectomy. Referrals may also be made for FP methods of choice that are not available at the time of need (for example, due to stockouts). We have defined the methods available directly and via referral and deleted referrals for family planning and retained the direct service delivery of FP in Table 1.
- **P.6: why was OPV included in the facility datasets given the focus on FP/RH services?** We initially collected OPV data because it was readily available and collected alongside other FP/RH data. We have deleted the OPV data from the datasets and graphs to remain consistent with the FP/RH theme.
- **P.6: You say the facility data is January – Dec 2020, but then later say you compare to 4 months prior to March 2020 – wouldn’t that include Nov and Dec 2019?** Data was not consistently collected across all facilities for November and December, so we have changed the text to read “2 months prior” in the paper and revised the graphs to ensure they include January 2020 – December 2020, reflecting two months prior to lockdowns. **• P. 6: Could you add a table that shows how many of each category of IDI respondent? Were all interviews conducted in English?** IDIs were conducted in English. A table has been added showing the break down between the three types of study participants.
  • **P.8: I’m a little confused – according to the Categories you mention, FP services were paused during Covid (“Category 2 patients were advised to stay at home unless non-preventive care was needed.) But then, later you say that provision of FP services “showed little effect’ – does this mean they ignored the guidance or those guidelines were not in fact implemented? Related to this, you mention what the policies were, but perhaps could be a bit more clear on how those policies were implemented**? We have clarified that FP services were “Category 2” but continued to be offered based on scheduled appointments to pre-plan the numbers seeking care at one time in order to reduce crowding and mitigate the risk of COVID-19 infection. Additionally, strict social distancing and triage measures were followed. We have clarified this in the paper. Additional detail on policies and how they were implemented related to FP has been added to the paper.
  **• P.9, last paragraph: you refer quite a bit to the triage and how that helped. Could you perhaps explain a bit more what this new triage was?** The triage system filters patients with symptoms of COVID-19 and sent them to specific clinicians for consultations in order to reduce contact with others. The waiting area where triage was held was open to allow proper ventilation, seating was organized to observe social distancing, and screening booths were used to reduce risk to COVID-19. A description of triage was added to the paper.
  • **P.14: “Some participants noted that ANC visits and facility-based delivery increased during COVID-19 because of the process of identifying people needing care in the community and referring them to the hospital.” Could you explain this statement a bit more? I’m not sure what “process” they refer to or how it changed?** During COVID community-based activities were conducted to communicate health risks and engage communities related to COVID. During these activities, health care workers also were able to refer pregnant women to delivery care as needed. This has been clarified in the paper.
  **• P. 17, last paragraph: You may want to cite supporting literature from the West African ebola epidemic that also showed FP visits rebounding after sharp decreases during the height of the epidemic.** We have added comparisons to the Ebola epidemic in FP/RH usage to the discussion section.
  **• Given that in many other places SRH services were highly impacted by Covid-19, could you say a bit more about why this was different?** Our results show an impact on SRH services during COVID followed by a rebound. This is consistent with other findings. It is also possible that the structured nature of delivering care within humanitarian settings made service delivery easier than in other locations or that the pre-existing challenges of delivering health care within humanitarian settings ensured that health care providers were already equipped to deal with challenging, shifting situations. Finally, the number of COVID cases within these areas was relatively low compared to other settings, prehaps reducing the impact of COVID-19. **• Can you say anything about how these programs will use these data to address gaps?** IHANN has used the adaptive methods that the programme utilized to inform Integrated Community Case Management (ICCM) for geriatrics and NCD cases. The adaptation of ICCM promoted early case detection and management not only for COVID-19 but also other common illnesses e.g. feverish illnesses, which is cost effective. This data has been shared at the virtual humanitarian coordination meetings held at the health facility, LGA and state level. These meetings were facilitated by UNHCR and FHI 360 to use the data to see the progress and challenges of program implementation and come up with solutions to the challenge. This has been added to the implications for programming section of the paper.

Reviewer #2: This manuscript presents a case study of how two projects being implemented in a humanitarian setting were able to adapt to ensure continuity of services during the COVID-19 pandemic. The article makes a great contribution to our understanding of how programs adapted during the pandemic. However, there are several issues that need to be addressed to improve the paper.

Title
**The title is not all-encompassing as it references FP/RH services, yet the article includes child immunization services.** We have deleted child immunization services from the manuscript, including graphs.

Abstract
**The structure of the abstract needs to be revised. Lessons learned ideally should come last. In the result section, it might be better to present the quantitative data first before the qualitative data.** We have changed the structure of the abstract accordingly.

Methods
**• Provide some background information on the health facilities where data was collected.** The data from the IHANN project consists of health facilities in a highly populated internally displaced persons camp in hard-to-reach rural areas of Borno state. The health facilities provide essential primary health care services. Due to the prolonged crisis in Northeast Nigeria, the facilities are mostly supported by NGOs. The data from the UNHCR project consists of public, government-run health facilities in host communities and camps. This information has been added to the paper.

• **Provide the rationale for restricting the analysis to a 4-month pre-COVID period. Are the 4 months adequate to illustrate the trend in FP service provision accounting for seasonality differences?** At the time of data collection, it was not feasible to collect data from before that time period. This lack of more extensive historical data is a limitation to the study, and this has been noted in the limitations section.

Results
• **Outline the 8 policies that were reviewed.** Additional detail about the policies has been added to page 8.
• **Add more verbatim quotations to illustrate the qualitative findings. For instance, in the COVID-19 adaptations-no qualitative quotes have been presented**. The section titled COVID-19 adaptation refers to all the qualitative results (infection prevention and control, workflow adaptations, providers’ roles, outreach services, and FP commodities). Additional quotes haven been added throughout the qualitative results.
• **For ease of reviewing the graphs, indicate when COVID began, and when services were adapted.** This was explained in paragraph 3 of the introduction to provide context regarding the timeline of COVID in Nigeria. As the national lockdown, state lockdown occurred at different times and the timeline of the service adaptation is not known precisely, we prefer to describe this in the text instead of the figures.
• **On the quantitative data, consider including the median (and IQR) number of attendances pre-COVID compared to during the COVID period and after.** Because it is difficult to distinguish between “during COVID” and “after COVID”, we decided not to compare attendances accordingly.
• **The qualitative results highlight the changes that were made without presenting what was happening before. For instance, IHANN staff worked two-week shifts, which was an adaptation but was happening prior to this change to help understand the changes that were made.** Before COVID 19, IHANN staff are based full time at the deep field sites continuously for six weeks followed by five days of recuperation outside of camps. At field sites, they share accommodation, dining areas, kitchen, washrooms. During COVID-19 staff were divided into small groups, who rotated on bi-weekly bases to and from deep field to limit the number of staff at each location and promote social distancing.
• **On family planning, the authors indicate that disruptions caused some patients to change methods to what was available. Provide more information on the contraceptive methods that were stocked out during this period.** In IHANN, contraceptive implants were unavailable at times. For UNHCR, there was minimal stockout because FHI 360 supported last mile delivery in Cross River state. This has been added to the paper.
• **In several instances, it is unclear who is referenced by the quotes. In many instances, the authors have indicated participants stated. Considering that the interviews were conducted among healthcare providers, project staff, and outreach workers from the two projects, it will be helpful if the authors could consistently indicate whose verbatim quotations are being referenced.** The type of participant has been added for every quote.

**Given the variability in the service uptake data between the two projects, was there an analysis of the qualitative data to help provide additional insights on what could have driven the differences considering all the study sites were from the humanitarian setting?** While both the qualitative data from both projects describe adaptations during COVID-19 to ensure the continuation of services, more key informants from IHANN II noted that they were unable to meet the needs of beneficiaries compared to UNHCR-SS-HNIR key informants. Given beneficiaries served by IHANN II are in highly populated camps in settings affected by conflict it seems likely that played a role in the difference seen between the two. (See also the response to the final question regarding difference between the two humanitarian projects.)

Limitation
**The limitation section should come at the end of the discussion section.**  The limitation section has been moved to come after the discussion.

Discussion
• **Several statements have been made without being referenced. For instance, the first sentence-FP/RH is a key and often unfulfilled need of women and girls in humanitarian settings.** We have added references to ensure all statements are referenced as needed.
• **Discussion section mostly paraphrases the results. There is little reference to existing literature to explain the findings.** Additional citations have been added to the discussion to contextualize the results.

• **Explain variability in the results presented. For instance, in the IHANN service trends, there was a reduction in the uptake of ANC services between April to June, but that same pattern is not replicated among pregnant women receiving HIV testing and counseling. FP services also seem unaffected.** We believe that HIV counseling and testing was able to increase once both projects received support from the Global Fund and PEPFAR partners providing HIV care and treatment to integrate HIV services with ANC care. Additionally, the IHANN II Project does prioritize HIV testing. Family planning services were relatively unaffected but there were fairly small numbers of women received these at all points in 2020. Regarding the decline in ANC services under IHANN II in April, we aren’t sure about the exact cause of this. Possible explanations include clients didn’t view ANC as a vital service early in the pandemic or initial challenges to provision of ANC during the early COVID-19 time period were later overcome as service delivery modifications were put into place. This has been added to the manuscript.

**Additionally, the IHANN trends also differ from UNCHCR service trends. Provide more insights on these differences considering that both data were from the humanitarian setting.** While both programs operate in humanitarian settings within Nigeria however, the impact of prolonged crisis and regional and community differences between the two is large. IHANN is implemented in North-East region and UNHCR project is implemented in South-South and the impact of the prolonged crisis in the two regions is quite different including the population, settlement, living conditions, culture, and religion among other contextual differences. IHANN II is implemented a highly populated internally displaced persons camp in hard-to-reach rural areas within an extremely fragile security situation. The IHANN II health facilities provide essential primary health care services, and the facilities are mostly supported by NGOs. UNHCR-SS-HNIR is implemented within public, government-run health facilities in host communities and camps under minimal security constraints. Given this, the adaptation to the prevailing security context in the middle of COVID was significantly different for IHANN and UNHCR. All of these factors may have contributed to the overall trends noted. This information has been added to the paper.
